# Development and pre-clinical evaluation of an isosorbide-based pit and fissure sealant achieving low polymerization shrinkage and high biocompatibility

**DOI:** 10.3389/fbioe.2026.1730749

**Published:** 2026-04-01

**Authors:** Ming Zhang, Yinan Cui, Su Yang, Xin Liu, Baiyan Sui, Jiao Sun

**Affiliations:** Shanghai Key Laboratory of Stomatology, Shanghai Ninth People’s Hospital, Shanghai Jiao Tong University School of Medicine, College of Stomatology, Shanghai Jiao Tong University, National Center for Stomatology, National Clinical Research Center for Oral Diseases, Shanghai, China

**Keywords:** bio-based sealant, caries prevention, biocompatibility, isosorbide, polymerization shrinkage

## Abstract

**Objectives:**

Resin-based sealants (RBSs) are widely used to prevent pit and fissure caries in clinical dentistry. However, their high density of C=C double bonds leads to volumetric shrinkage during photo-polymerization, which may compromise enamel bonding. Additionally, the biosafety of commonly used monomers such as bisphenol A glycerolate dimethacrylate (bis-GMA) and triethylene glycol dimethacrylate (TEGDMA) remains a concern due to potential health risks in adolescent patients. This study aimed to develop a bis-GMA- and TEGDMA-free pit and fissure sealant to address these limitations.

**Methods:**

A novel family of isosorbide-derived dental sealants (ISDSs) was developed using a synthesized isosorbide-based matrix, bis(2-(methacryloyloxy)ethyl) disuccinate isosorbide (IBMEDS), and a diluent, bis(2-methylacrylate) isosorbide (IBM). The performance of ISDS was evaluated against the commercial Clinpro™ sealant in terms of polymerization shrinkage, cytotoxicity, physicochemical properties, microleakage resistance, and stability of the sealed area under artificial aging conditions.

**Results:**

Compared with Clinpro, ISDS exhibited a 34% reduction in polymerization shrinkage and a more than sevenfold increase in L929 cell viability, attributed to its bio-based structure and low double-bond density. Moreover, ISDS demonstrated superior physicochemical properties, including higher shear bond strength, more effective marginal adaptation to enamel, lower water absorption and solubility, and more sustained and stable long-term fluoride release, while maintaining comparable microleakage resistance.

**Conclusion:**

A fully bio-based, fluoride-containing isosorbide-derived dental sealant (ISDS) was developed to overcome the polymerization shrinkage and biocompatibility limitations of conventional resin-based sealants. This novel sealant offers enhanced long-term stability, excellent biocompatibility, improved marginal adaptation, and sustained fluoride release, representing a safer and more effective alternative for caries prevention in children and adolescents.

## 1 Introduction

Pit and fissure sealants are the most effective means of protecting pits and fissures on occlusal tooth surfaces from caries, and these sealants are prevalently utilized among children and teens under the age of 13. Resin-based sealants (RBSs) are the most commonly used in clinical practice for the prevention of caries and can reduce the occurrence of caries in the first molars by more than 50% (Ahovuo-Saloranta et al., 2013). However, more than 80% of the components of RBSs are organic monomers, such as bisphenol A glycerolate dimethacrylate (bis-GMA), urethane dimethacrylate (UDMA), and triethylene glycol dimethacrylate (TEGDMA), which possess a high density of C=C double bonds, contributing to a significant amount of volumetric shrinkage during photopolymerization, thus weakening the bond strength (BS) between the material and the enamel, even leading to the formation of marginal gaps. Another notable drawback is the intrinsic cytotoxicity of these monomers in RBSs, which arises from the release of monomers into the oral environment due to incomplete photopolymerization and biological degradation of the resin material, leading to the release of monomers into the oral environment. As children and adolescents are the primary target groups for RBS applications, biosafety challenges posed by monomer components cannot be overlooked (Kurt et al., 2018; Becher et al., 2018). Therefore, the key to reforming these two drawbacks, namely, polymerization shrinkage (PS) and bio-toxicity, is to construct an entirely novel resin-based matrix system that is biocompatible and demonstrates low volumetric shrinkage, consequently improving the RBS effect of caries prevention and fundamentally protecting the health of adolescent patients.

Isosorbide, which is produced from a wide range of renewable biological resources such as sucrose, starch, and glucose, is a bio-based bi-heterocyclic diol that is qualified by the Food and Drug Administration (FDA) of the U.S. as “generally recognized as safe” and has potential application value for biocompatible materials (Łukaszczyk et al., 2012; Zhang et al., 2013; Gao et al., 2018). Several studies have shown that polymers containing isosorbide can be used as biomedical engineering materials and exhibit excellent biocompatibility, promoting cell adhesion, proliferation, and differentiation (Park et al., 2013; Gregorí Valdés et al., 2018). Jun et al. (2020) reported a new isosorbide-core dimethacrylic monomer, ISDB, and its RBS with a high diluent (TEGDMA) content (ISDB: TEGDMA = 29.8/70.2 (wt%)) showed no adverse estrogenicity, which is usually caused by bisphenol A (BPA), as BPA was not generated as a hydrolytic byproduct from ISDB. However, because a high amount of TEGDMA diluent was added (in commercial RBS, the ratio of the matrix to diluent is at least 50/50 (wt%)), the amount of C=C double bonds in this RBS was further increased; thus, the risk of curing shrinkage and endodontic cytotoxicity generated by the monomer leakage from the resin after polymerization was aggravated. Therefore, to reduce these risks and application drawbacks, it is necessary to first analyze a low-viscosity matrix and select a diluent with an appropriate bio-based molecular structure and then resolve the bottleneck regarding the volumetric polymerization shrinkage and toxicity of the current RBS *via* the low C=C double bond density and bio-based structure of the matrix and the diluent monomers.

Based on the aforementioned academic theory, we innovatively designed this study and constructed a new family of an isosorbide-derived dental sealant (ISDS), in which the designed monomer bis(2-(methacryloyloxy) ethyl) disuccinate isosorbide (IBMEDS), synthesized with isosorbide and 2-methacryloyloxyethyl succinic acid, was used as the matrix and bis(2-methylacrylate) isosorbide (IBM) was used as the diluted monomer. We also characterized a series of physicochemical properties of this ISDS, including fluidity, enamel wettability, C=C double-bond conversion rate, and hydrophilicity after curing, and evaluated its cytotoxicity. Moreover, we analyzed the application performance, including polymerization shrinkage, shear bond strength, water sorption (WS) and solubility (SL), fluoride release behavior, and microleakage, to provide a critical scientific basis for the clinical application of this ISDS.

## 2 Materials and methods

### 2.1 Materials

Isosorbide, camphorquinone (CQ), tetrabutylammonium tetrafluoroborate, 1-(3-dimethylaminopropyl)-3-ethylcarbodiimide hydrochloride (EDC.HCl), and 3-methacryloxypropyltrimethoxysilane (γ-MPS) were purchased from Beijing InnoChem Science & Technology Co., Ltd. (Beijing, China). 2-(Dimethylamino)ethyl methacrylate (DMAEMA) and mono[2-[(2-methyl-1-oxo-2-propenyl)oxy]ethyl]ester were provided by Shanghai Titan Scientific Co., Ltd. (Shanghai, China). Methacryloyl chloride was obtained from Shanghai Aladdin Biochemical Technology Co., Ltd. (Shanghai, China). Anhydrous dichloromethane (CH_2_Cl_2_) and chloroform-D (CDCl_3_, with tetramethylsilane as the internal standard) were purchased from Shanghai J&K Scientific Co., Ltd. (Shanghai, China). Bisphenol A-glycerolate dimethacrylate (bis-GMA) was purchased from Sigma-Aldrich (Shanghai, China). Triethylene glycol dimethacrylate (TEGDMA) was provided by Shanghai Macklin Biochemical Co., Ltd. (Shanghai, China). 4-Dimethylaminopyridine (DMAP) was obtained from GL Biochem Ltd. (Shanghai, China). The inorganic dental glass fillers (0.7 μm) were obtained from Donghai Colorful Mineral Products Co., Ltd. (Lianyungang, China). The 3M ESPE Clinpro™ Sealant (3M Company, Shanghai, China) was used as the control group. The IBMEDS and IBM monomers were synthesized in our laboratory and used as the matrix and diluent, respectively. The dental glass fillers were then treated with γ-MPS. All the other chemicals and solvents were used as received, without further purification.

### 2.2 Synthesis of isosorbide bis(2-(methacryloyloxy)ethyl) disuccinate

EDC.HCl (23.61 g, 0.123 mol), triethylamine (24.92 g, 0.246 mol), mono[2-[(2-methyl-1-oxo-2-propenyl)oxy]ethyl]ester (23.63 g, 0.102 mol), and 500 mL CH_2_Cl_2_ were added to a 1,000 mL round-bottom flask. Isosorbide (5 g, 0.034 mol) and DMAP (0.84 g, 0.0068 mol) were added to the aforementioned suspension. The mixture was then stirred and heated for 20 h, and the color of the mixture turned dark yellow. After washing with water and a NaHCO_3_ saturated solution, the mixture was dried with Na_2_SO_4_, and the solvent was evaporated. The residue was purified by silica column chromatography (eluent: *n*-hexane/CH_2_Cl_2_/methanol = 100/400/6–100/400/10) to obtain 13.82 g (71%) of the IBMEDS monomer as a transparent slightly yellowish liquid. The structure of the IBMEDS was confirmed *via* ^1^H nuclear magnetic resonance (^1^H NMR) and low-resolution mass spectrometry (LR-MS).

**FIGURE 1:**
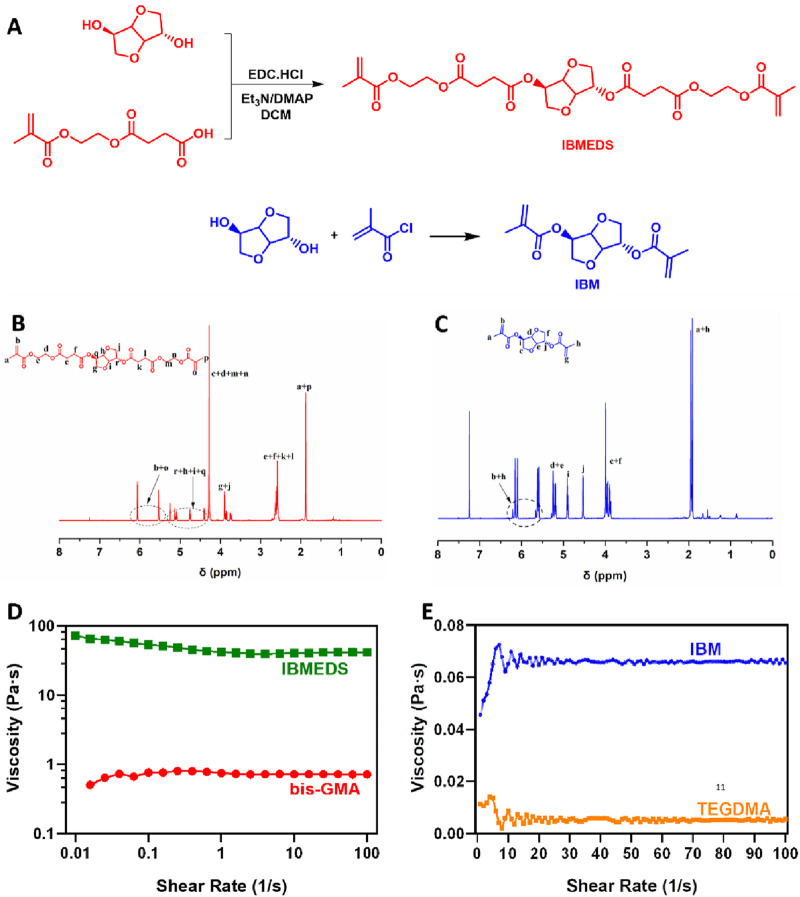
Synthesis and characterization of the IBMEDS matrix monomer and IBM diluent monomer. **(A)** Synthesis scheme of the IBMEDS matrix and IBM diluent monomer. ^1^H NMR spectrum of the **(B)** IBMEDS and **(C)** IBM molecules. Measured viscosities of the **(D)** IBMEDS matrix monomer and bis-GMA and the **(E)** IBM and TEGDMA diluent monomers.

^1^H NMR (JEOL ECZ 400 YH, 400 MHz, CDCl_3_; Figure 1B): *δ* (ppm): 6.06 (s, 2 H), 5.53 (d, 2 H), 5.10 (m, 2 H), 4.75 (t, 1 H), 4.40 (d, 1 H), 4.28 (s, 8 H), 3.83 (m, 4 H), 2.62 (m, 8 H), and 1.88 (s, 6 H).

LR-MS (ESI+, Shimadzu AXIMA Performance, Japan; Supplementary Figure S1a): the calculated *m/z* of C_26_H_34_O_14_ was 570.54; the found values were 593.2 [M + Na]^+^ and 588.3 [M + NH_4_]^+^.

### 2.3 Synthesis of isosorbide bis(2-methylacrylate)

Isosorbide (5.01 g, 0.034 mol), triethylamine (14.56 g, 0.144 mol), and DMAP (0.42 g, 3.4 mmol) were dissolved with 30 mL of anhydrous CH_2_Cl_2_ in a 250-mL round-bottom flask, which was followed by immersing the flask in a 0 °C ice-water bath. Methacryloyl chloride (11.92 g, 0.114 mol) was then added dropwise to the above mixture. The reaction mixture was then stirred overnight at room temperature. Subsequently, 50 mL of water was added to the mixture. The organic layer was then extracted and dried using Na_2_SO_4_. After evaporation of the solvent, the residue was purified by silica column chromatography (eluent: *n*-hexane/CH_2_Cl_2_/methanol = 100/300/5) to obtain 5.14 g (54%) of IBM as a transparent colorless liquid.

^1^H NMR (JEOL ECZ 400 YH, 400 MHz, CDCl_3_; Figure 1C) *δ* (ppm): 6.13 (d, 2 H), 5.60 (d, 2 H), 5.21 (m, 2 H), 4.90 (t, 1 H), 4.53 (d, 1 H), 3.94 (m, 4 H), 1.95 (s, 3 H), and 1.91 (s, 3 H).

LR-MS (ESI+, Shimadzu AXIMA Performance, Japan; Supplementary Figure S1b): the calculated *m/z* of C_14_H_18_O_6_ was 282.29; the found values were 283.1 [M + H]^+^, 300.1 [M + NH_4_]^+^, and 305.0 [M + Na]^+^.

### 2.4 Silane-modification of inorganic glass fillers

The dental glass fillers were silanized with γ-MPS according to a previously reported method (Chen and Brauer, 1982). The glass powder (10 g) and γ-MPS (1 g) were dispersed into 50 mL of 60% ethanol, which had a pH of 3–4. The mixture was initially stirred at room temperature for 1 h, followed by additional stirring at 70 °C for an additional 1 h. Subsequently, the mixture was evaporated at 65 °C to remove the solvent, and the powder was then heated at 120 °C for 2 h. The silane-modification results of SiO_2_ (Supplementary Figure S2) were confirmed using an FT-IR spectrometer (Thermo Fisher Scientific, NICOLET iS5, United States).

### 2.5 Preparation of the pit and fissure sealants

The commercially available dental sealant 3M ESPE Clinpro™ Sealant (Clinpro), which consists of bis-GMA and TEGDMA as the main components, was selected as the control material. The synthesized monomers IBMEDS and IBM were used as polymerization monomers for our isosorbide-based dental sealant (ISDS), respectively. CQ and DMAEMA were used as the photo-initiator system. The ratios of the polymerization monomers, inorganic glass powder, and CQ and DMAEMA were 89 wt%, 10 wt%, 1 wt%, respectively. The filler contents of 3–4 types of commercial dental sealants were used as a reference to determine the amount of glass powder added to our ISDS. Three groups of ISDSs with different compositional ratios and three groups of fluoride-containing ISDSs were prepared, as listed in Supplementary Tables S1, S2, respectively. All the components were mixed using a SpeedMixer (FlackTek DAC 330-100 SE, United States).

### 2.6 Measurement of rheological viscosity

The rheological viscosity measurements of bis-GMA, TEGDMA, IBMEDS, IBM, 3M Clinpro, and ISDSs were performed using a rheo-viscometer with a plate geometry (Anton Paar MCR 302, Austria). The samples were measured at 37 °C, and the shear rate ranged from 10^–2^ to 10^2^ s^-1^.

### 2.7 Measurement of the contact angle

A contact angle meter (Kino SL250, United States) was used to measure the following two types of contact angles at room temperature.

The contact angle of sealants on bovine enamel: A drop of the sealant mixture without photo-irradiation was placed on a bar of bovine enamel, and the contact angle was measured. Five pieces of bovine enamel were used for Clinpro and each ISDS formulation.

The water contact angle on a sealant disc: A disc specimen (15 mm diameter × 1 mm thickness) was prepared by light-curing the corresponding sealant mixture in a metal mold for 40 s. A drop of distilled water was placed on a small disk to measure the water contact angle. For each sealant formulation, five discs were prepared, and three areas on each disc were selected for this measurement.

### 2.8 Measurement of double-bond conversion

The degree of double-bond conversion (DC) of Clinpro and ISDSs was measured using an FT-IR spectrometer with an attenuated total reflectance (ATR) accessory (Thermo Fisher Scientific, NICOLET iZ10, United States). The IR spectrum of the sample without blue light exposure was obtained by scanning the sealant on top of a potassium bromide (KBr) disk. Photopolymerization was then conducted by irradiating a drop of the sealant sample with blue LED light (430 nm–480 nm wavelengths, 1,470 mW/cm^2^ intensity, 3M ESPE Elipar™ DeepCure-S, United States) for 20 s to obtain a sample disc (15 mm diameter × 1 mm thickness), which was scanned directly, and the spectrum of the sealant after photopolymerization was recorded. The degree of double-bond conversion was determined by calculating the ratio of the methacrylate C=C peak area to the C=O peak area before and after photopolymerization according to Equation 1 as follows:

DC=1−AC=C/AC=OpAC=C/AC=O0×100%, (1)

where A_C=C_ and A_C=O_ are the absorbance intensities of methacrylate C=C at 1,636 cm^-1^ and carbonyl C=O at 1,720 cm^-1^, respectively; (A_C=C_/A_C=O_)_0_ and (A_C=C_/A_C=O_)_p_ represent the normalized absorbance of the functional groups before and after photopolymerization, respectively. Five trials were conducted for each sealant formulation.

### 2.9 Assessment of cell viability

An unpolymerized liquid sample was obtained from each sealant formulation group and placed in a cylindrical mold (8 mm diameter × 4 mm thickness). All the samples were light-cured for 40 s using a blue LED light (430 nm–480 nm wavelengths, 1,470 mW/cm^2^ intensity, 3M ESPE Elipar™ DeepCure-S, United States) to obtain the corresponding cured specimens. Six samples were prepared from each treatment group. The specimens were immersed in Dulbecco’s modified Eagle medium (DMEM, Gibco, United States) containing 10% fetal bovine serum (FBS) and 100 U/mL penicillin and streptomycin (PS) at a ratio of 0.2 g/mL, and they were cultivated in a 37 °C constant-temperature cell incubator for 24 h; the conditioned culture medium was obtained *via* filtration. L929 cells were logarithmically plated, exposed to different conditioned media for 24 h, and measured using the Cell Counting Kit-8 (CCK-8; Dojindo).

### 2.10 Immunofluorescence assay

L929 cells were cultured in 12-well plates using the method described in Section 2.2.8. Immunofluorescent staining was performed after 24 h of incubation with the respective elution solutions for each group. The cells were fixed with 4% paraformaldehyde (PFA) for 30 min, permeabilized with 0.25% Triton X-100 (Sigma-Aldrich) in PBS for 3 min, and non-specific binding sites were blocked with 8% bovine serum albumin (BSA) for 1 h. The cells were subsequently incubated with 5 μg/mL of mouse monoclonal anti-human fibroblast surface protein (FSP) antibody (Abcam, Cambridge, United Kingdom) for 1 h. After washing, the cells were incubated with Alexa Fluor 488 anti-mouse IgM and DAPI counterstains, observed, and photographed under a fluorescence microscope (Cytation 3 Imaging Reader, BioTek, Winooski) at an excitation wavelength of 488 nm and emission wavelength of 525 nm.

### 2.11 Measurement of polymerization shrinkage

P during the photo-curing reaction was evaluated using the laser-ranging method provided by YY/T 1599–2018. An unpolymerized sealant was added to a cylindrical mold (8 mm diameter × 4 mm thickness), followed by blue-light irritation for 1 min. After curing, data regarding the displacement of the sample edge were obtained and recorded as L. The linear polymerization shrinkage rate was calculated using Formula 2.

$$\text{PS}=\frac{L}{R}\times 100\%,$$ (2)

where R is the radius of the mold; in this experiment, R = 4 mm.

Six trials were performed for each sealant formulation.

### 2.12 Measurement of the shear bond strength of the enamel

A total of 10 caries-free third molars were collected for testing the shear bond strength of the enamel. All crowns were divided into mesial and distal surfaces using a precision automatic micro-cutting machine (Mecatome T210, Presi). The crowns were embedded in self-curing resin to expose their intact mesial or distal surfaces, thus forming 20 tooth surfaces. Each enamel surface was polished with 4,000-grit alumina paper under running water for 30 s, rinsed with water spray, and dried. All the enamel surfaces were etched with 35% H_3_PO_4_ gel for 30 s, rinsed with an air–water spray for 30 s, and dried for 10 s. A total of 20 polytetrafluoroethylene molds with an inner diameter of 3 mm were vertically placed on the prepared tooth surfaces; the edges of the molds and tooth surfaces were sealed with wax. Two groups of unpolymerized sealants were placed into the molds along the inner wall and cured for 40 s under blue LED light (430 nm–480 nm wavelengths, 1,470 mW/cm^2^ intensity, 3M ESPE Elipar™ DeepCure-S, United States). After curing, the molds were removed, and the diameter of each cylinder was measured using a Vernier caliper. A thermal cycling machine (TC-501F, Weier) was used to artificially age all the samples, which were cycled 5,000 times in cold (5 °C) and hot (50 °C) water for 30 s each. A universal testing machine (EZ20, LLOYD) was used to apply a shear load at a crosshead speed of 1 mm/min parallel to the bonding interface until failure occurred. The shear bond strength of each group of samples was calculated using Formula 3:

$$\text{BS}=\frac{4P}{\;\left(\pi d^{2}\right)},$$ (3)

where P is the load at the time of failure and d is the diameter of the bonding area of the sealant on the tooth surface.

### 2.13 Measurement of water sorption and solubility

The WS and SL of the sealant specimens were measured according to the specifications provided by ISO Standard 4049. The unpolymerized sealant was added to a steel mold, followed by blue light-curing for 40 s to prepare a disc specimen (15 mm diameter × 1 mm thickness). The specimens were freeze-dried to remove humidity and then weighed to an accuracy of 0.1 mg until the mass (m_1_) became constant. Subsequently, the specimens were immersed in distilled water at 37 °C for 7 days, after which they were removed from the water and weighed to obtain the mass (m_2_). Finally, the specimens were freeze-dried once again until a constant mass was obtained (m_3_). The WS and SL values of the specimens were calculated using Formulas 4, 5, respectively:

$$\text{WS}=\frac{\left(m_{2}-m_{3}\right)}{V},$$ (4)

$$\text{SL}=\frac{\left(m_{1}-m_{3}\right)}{V},$$ (5)

where V is the volume of the specimen.

Five disc-shaped specimens were prepared to perform the measurement for each sealant formulation.

### 2.14 Measurement of microleakage

A total of 20 healthy third molars were collected, washed under running water, and cleaned up with a toothbrush. The enamel surface was then etched with a 37% H_3_PO_4_ gel for 30 s, rinsed with distilled water for 20 s, and air-dried for 15 s. The unpolymerized sealant mixture was applied on the occlusal surface of the molar using a microbrush and exposed to blue light for 20 s to obtain the sealant–enamel specimen. A total of 10 specimens of each sealant formulation were prepared.

The specimens were then subjected to aging treatment with 5,000 thermal cycles at temperatures ranging between 5 °C and 55 °C, followed by brushing nail varnish onto the sealant layer. The specimens were immersed in a 2% methylene blue solution at 37 °C for 24 h and mesiodistally sectioned using a precision automatic micro-cutting machine (Mecatome T210, Presi). A stereomicroscope (SteREO Discovery. V12, Zeiss) was used to determine the penetration depth of the dye at a magnification of ×30, which was ranked in the following levels:

1. No dye penetration.
2. Dye penetration limited to the outer half of the sealant.
3. Dye penetration extending to the inner half of the sealant.
4. Dye penetration extending to the underlying fissure.

### 2.15 Scanning electron microscopy

Two caries-free third molars were collected to observe the enamel–sealant interface using SEM. After pit and fissure sealant treatment for each tooth, a precision automatic microtome (Mecatome T210, French company Presi) was used to cut hard tissue slices (approximately 2 mm thick) from the corresponding areas on the buccal and lingual sides of each tooth. Each sample was then polished with a 4,000-grit aluminum oxide sandpaper under running water. The samples were etched with 35% H_3_PO_4_ gel for 30 s and briefly deproteinized with a 2% NaClO solution. After rinsing with distilled water, the samples were dried and sputter-coated with gold. The surface morphology was observed using field-emission SEM (FE-SEM; JSM-7800F, JEOL) at magnifications of ×500 or 1,000×.

### 2.16 Measurement of microgap

Five caries-free third molars were collected for the microgap measurements in each sealant group. After the pit and fissure sealant treatment for each tooth, the precision cutting machine (Mecatome T210, Presi) was used to cut all the tooth crowns into two pieces along the buccal–lingual direction, producing a total of 10 hard-tissue slices. All the slices were gold-coated and examined via SEM (JSM-7800F, JEOL). A 2 mm area was selected at the center of the fissure, and the widest microgap width between the sealant and enamel surface was measured using a scanning electron microscope at a magnification of 1,000×.

### 2.17 Measurement of fluoride releasing

The concentration of fluoride released was measured using ion chromatography (Thermo Fisher Scientific, DIONEX AQUION, United States). Cylindrical specimens (∼6 mm diameter × 3 mm thickness) prepared by light-curing the liquid sealant mixture in a plastic mold for 60 s were immersed in distilled water at 37 °C for 3, 7, 14, 28, and 124 days. The aqueous extracts were collected at the end of the immersion period and injected into the ion chromatograph to calculate the fluoride concentration in each leaching solution. The concentration of fluoride was determined using the linear calibration curve as the reference, which was plotted based on the relationship between the peak intensity at its retention time and the concentration of the standard sample (GBW(E)082682, TMRM®) at 0.3, 0.5, 1, 2, 5, 10, 20, 30, and 50 μg/mL. Three specimens of each sealant formulation were prepared.

### 2.18 Statistical analysis

Statistical analysis was performed with SPSS 26.0 software for WINDOWS (IBM, United States). Data were analyzed with one-way ANOVA and an independent samples t-test when the normal distribution and equality of variance were met. Statistical significance was accepted at *p* < 0.05. The values are expressed as the mean ± SD of at least three independent experiments.

## 3 Results

### 3.1 Characterization of IBMEDS and IBM

Light-curable IBMEDS and IBM were synthesized as alternative monomers of bis-GMA and TEGDMA, respectively. After column chromatography purification, the chemical structures were characterized by ^1^H NMR and ESI-MS (Figures 1B,C and Supplementary Figures S1A,B). The viscosity of IBMEDS was measured as 0.748 Pas (Figure 1D), which is much lower than that of bis-GMA (42 Pas) at the condition of 37 °C and shear rate of 1 s^-1^. Meanwhile, the monomer IBM displayed a viscosity of 0.046 Pas (Figure 1E), which is slightly higher than that of TEGDMA (0.011 Pas).

### 3.2 Fabrication and preference of ISDS

**FIGURE 2:**
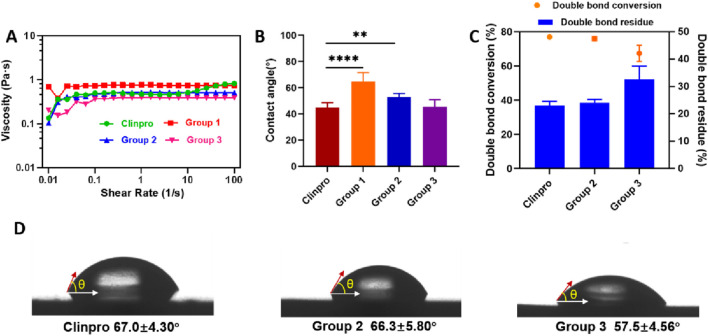
Screening of ISDS: comparison of Clinpro and groups 1–3. **(A)** Rheological viscosities. **(B)** Contact angle on bovine enamel. **(C)** Double-bond conversion and residue rate (%). **(D)** Water contact angle on discs. Error bars represent SD. **p < 0.01; ****p < 0.0001.

Three groups of ISDS containing 89 wt%, 75.65 wt%, and 62.3 wt% of IBMEDS were recorded as groups 1, 2, and 3, respectively. The order of viscosity value exhibited by the ISDS group 1–3 and Clinpro was group 1 > group 2 ≈ Clinpro > group 3 at the shear rate range of 0.1 s^-1^–10 s^-1^ (Figure 2A). The contact angles of the uncured ISDS groups 1 and 2 on bovine enamel were calculated as 64.7° ± 6.86° and 52.8° ± 2.92°, respectively, which are significantly higher than that of Clinpro (44.8° ± 3.98°) (*p* < 0.01). ISDS group 3 showed the contact angle value of 45.6° ± 5.22°, which was comparable with that of Clinpro (*p* > 0.05), as shown in Figure 2B. After photopolymerization, ISDS group 2 and Clinpro displayed a C=C double-bond conversion rate of 75.9% ± 1.20% and 76.9% ± 1.52%, respectively, and their corresponding C=C double-bond residue rate was 24.1% ± 1.20% and 23.0% ± 1.52%, respectively. Neither the C=C conversion rate nor the residue rate demonstrated a significant difference between ISDS group 2 and Clinpro (*p* > 0.05). The ISDS group 3 exhibited lower C=C conversion rate (67.3% ± 4.79%) and higher C=C residue rate (32.7% ± 4.79%) than Clinpro, and the difference was statistically significant (*p* < 0.001) (Figure 2C). The water contact angle on the surface of cured ISDS groups 2 and 3 and Clinpro were 66.3° ± 5.80°, 57.5° ± 4.56°, and 67.0° ± 4.30°, respectively. There was a significant difference between ISDS group 3 and Clinpro (*p* < 0.0001) (Figure 2D).

### 3.3 Cytotoxicity evaluation of the fluoride-containing ISDS

**FIGURE 3:**
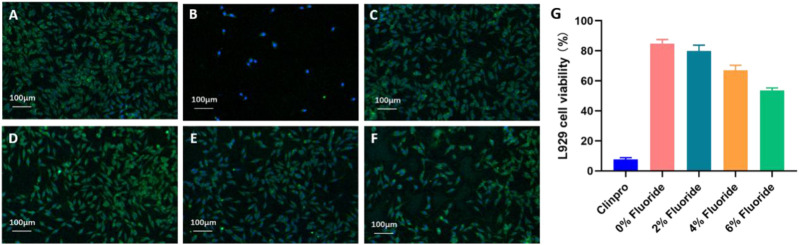
Cytotoxicity of the ISDS group 2 with different contents (wt%) of fluoride reagents and Clinpro. Fluorescence microscopy images: **(A)** blank control, **(B)** Clinpro, **(C)** 0% fluoride, **(D)** 2% fluoride, **(E)** 4% fluoride, **(F)** 6% fluoride, and **(G)** a histogram of the L929 cell viability. Error bars represent SD.

Cell viability of each ISDS with tetrabutylammonium tetrafluoroborate, the fluoride-releasing reagent, was higher than that of Clinpro (Figures 3A–F). The ISDS containing 0%, 2%, 4%, and 6% tetrabutylammonium tetrafluoroborate showed cell viabilities of 84.9% ± 2.63%, 79.9% ± 3.83%, 67.1% ± 3.21%, and 53.7% ± 1.59%, respectively, whereas that of Clinpro was only 7.69% ± 1.16%. There was a significant difference among these groups (*p* < 0.0001) (Figure 3G).

### 3.4 Long-term stability estimation of fluoride-containing ISDS

**FIGURE 4:**
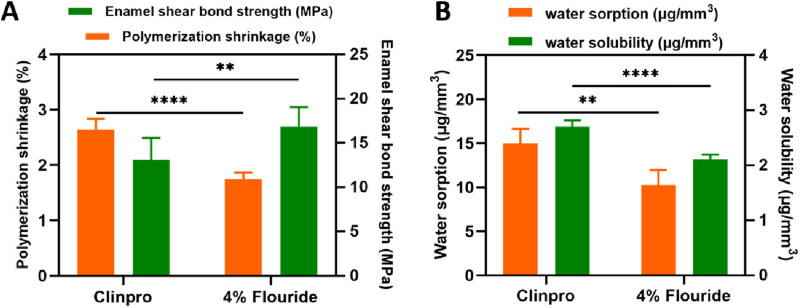
Long-term stability of the 4% fluoride-containing ISDS and Clinpro. **(A)** Linear PS and shear BS. **(B)** WS and SL. Error bars represent SD. **p < 0.01; ****p < 0.0001.

The 4% fluoride-containing ISDS showed much lower polymerization shrinkage and higher enamel shear bond strength, and the difference was statistically significant (*p* < 0.01). The photo-curing PS of 4% fluoride-containing ISDS and Clinpro was 1.74% ± 0.12% and 2.64% ± 0.20%, respectively, and their shear BS was 16.84 ± 2.22 MPa and 13.10 ± 2.45 MPa, respectively, after curing (Figure 4A). The 4% fluoride-containing ISDS exhibited 10.26 ± 1.68 μg/mm^3^ of WS and 2.11 ± 0.08 μg/mm^3^ of SL, while Clinpro showed higher WS and SL, which was 15.00 ± 1.66 μg/mm^3^ and 2.70 ± 0.12 μg/mm^3^, respectively (Figure 4B). There was a significant difference in both WS and SL between the two groups (*p* < 0.01).

### 3.5 Microleakage and enamel marginal adaptation studies of fluoride-containing ISDS

**FIGURE 5:**
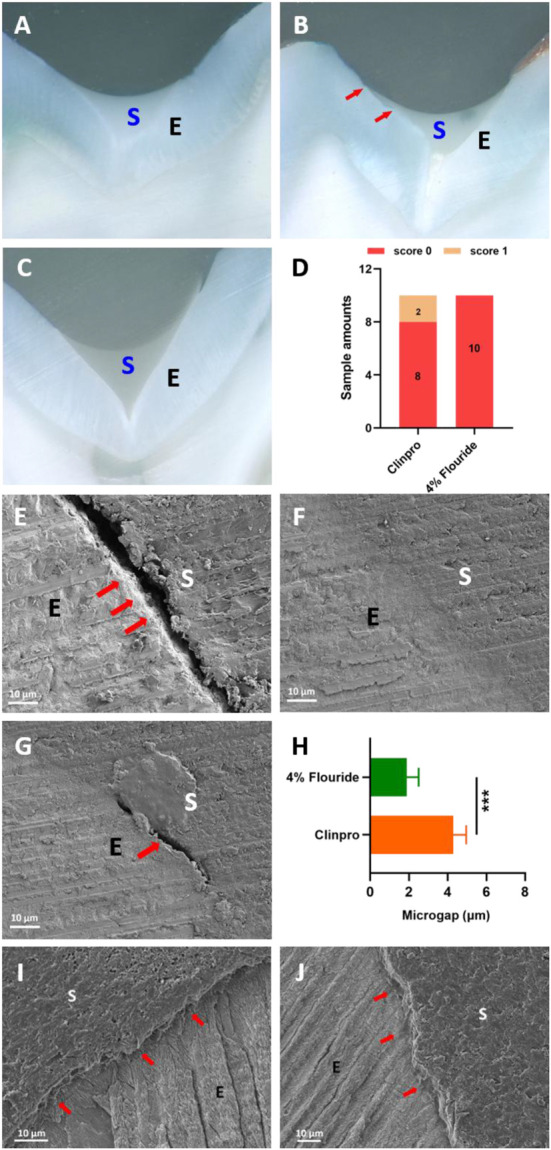
Post-sealing performance of the 4% fluoride-containing ISDS and Clinpro. Stereomicroscope images presenting the microleakage of **(A)** Clinpro, score 0; **(B)** Clinpro, score 1; and **(C)** ISDS group, score 0. **(D)** Statistical results of the microleakage scores. SEM images presenting the bonding surface of **(E)** Clinpro with a large microgap, **(F)** ISDS group with a continuous interface, and **(G)** ISDS with a narrow microgap. **(H)** Statistic width of microgaps. SEM image demonstrating the permeability of **(I)** Clinpro with sparse resin tags and the **(J)** ISDS with long and deep resin tags. Error bars represent SD. ***p < 0.001.

After sealing with two groups of sealants, no microleakage occurred in all the specimens of the 4% fluoride-containing ISDS (score 0), while in the Clinpro group, only two specimens exhibited dye penetration (score 1), and the difference was not statistically significant between the ISDS and Clinpro (*p* > 0.05) groups (Figures 5A–D; Supplementary Table S3). According to SEM image, the 4% fluoride-containing ISDS formed a more continuous interface than Clinpro and showed microgaps with a width of 1.89 ± 0.68 μm, which was much smaller than that of the Clinpro group, which was 4.29 ± 0.75 μm, and the difference was statistically significant (*p* < 0.001) (Figures 5E–H). In the ISDS group, long and deep resin tags were formed *via* uncured sealant liquid that penetrated into the enamel pores (Figure 5J), whereas only sparse resin tags were formed by Clinpro and inserted into the demineralized enamel pores (Figure 5I).

### 3.6 Fluoride-releasing behavior studies of fluoride-containing ISDS

During the first 14 days, the concentration of fluoride ions released by ISDS was obviously lower than that released by Clinpro, and the difference was statistically significant (*p* < 0.0001). After immersing two groups of specimens into distilled water for 3, 7, and 14 days, fluoride ion concentrations of ISDS were 0.56 ± 0.05, 1.41 ± 0.07, and 2.32 ± 0.25 μg/mL, respectively, and those of Clinpro was 1.84 ± 0.06, 3.62 ± 0.14, and 6.42 ± 0.70 μg/mL, respectively. After 28 days, fluoride ion concentrations in the ISDS and Clinpro groups were close, which were 11.5 ± 0.61 and 12.7 ± 1.01 μg/mL, respectively. After 128 days, the concentration of fluoride ions released by ISDS was 34.3 ± 2.65 μg/mL, much higher than that of the Clinpro group, which was 19.8 ± 0.68 μg/mL, and the difference was statistically significant (*p* < 0.001).

## 4 Discussion

RBSs are widely used for the prevention of pit and fissure caries; however, the controversial polymerization shrinkage and biocompatibility remain clinical challenges requiring prompt solutions. In this study, we improved the traditional structural model of the RBS, the bis-GMA/UDMA/TEGDMA system, and constructed an ISDS prepared with the synthesized isosorbide-cored matrix IBMEDS and diluent IBM. Because of its outstanding biocompatibility and low polymerization shrinkage, this ISDS is expected to remarkably improve and solve the clinical bottleneck problems of traditional RBSs.

### 4.1 Research of ISDS

#### 4.1.1 Fabrication of ISDS

For pit and fissure sealants, the sealing effect is a critical performance parameter that is closely related to the volumetric shrinkage of the materials during polymerization and directly affects the occurrence of microleakage and secondary caries and the effectiveness of the treatment. Moreover, the matrix component, viscosity, and C=C double-bond density are critical factors that determine the extent of polymerization shrinkage and the sealing effect. The traditional bis-GMA/UDMA/TEGDMA system is the main constituent of current commercial RBS products and accounts for more than 80% of the weight of the RBS. For the convenience of clinical operations, a large amount of dimethacrylic acid containing diluted reagent, TEGDMA, was added because of the high viscosity of the bis-GMA-like matrix, thus increasing the amount of C=C double bonds and aggravating the curing volumetric shrinkage of the RBS. This study first considered the design of a low-viscosity matrix with bio-structured isosorbide as the core that can be used to replace bis-GMA, thus constructing a precondition for reducing the proportion of the diluent added (Łukaszczyk et al., 2012; Shin et al., 2017). Furthermore, because of the incomplete curing and long-term hydrolytic degradation after polymerization, the unreacted monomers including bis-GMA, UDMA, or TEGDMA and their corresponding derivatives dissolve easily from the RBS materials, posing potential threats to the safety of the dental pulp cells, fibroblasts, and the reproductive and developmental system of humans (Rochester, 2013; Rubin, 2011; Wisniewska-Jarosinska et al., 2011), especially the health of children and teens who are the main group for which RBSs are applied. A diluent monomer with a rigid core was designed as an alternative to traditional TEGDMA to limit the mobility and leakage of residual monomers in cured resin materials.

Based on the aforementioned designed ideas, 2-methacryloyloxyethyl succinic acid and methacryloyl chloride were reacted with isosorbide to obtain the IBMEDS matrix monomer and IBM diluted monomer (Figure 1A); both of their chemical structures were determined using ^1^H NMR and ESI-MS (Figures 1B,C and Supplementary Figures S1A,B). Moreover, according to the rheological test results, the viscosity of IBMEDS was significantly lower than that of bis-GMA under the same measurement conditions (Figure 1D), which can be ascribed to the lower mechanical strength of IBMEDS and the non-existing interaction of hydrogen bonds between the molecules compared with that in bis-GMA (Moszner et al., 2008a; Alrahlah et al., 2021). Additionally, another monomer, IBM, exhibited slightly higher viscosity than that of TEGDMA but still showed favorable fluidity (Figure 1E). Based on the construction strategy of the RBS with low double-bond density as previously indicated, the synthesized IBMEDS and IBM were blended in different ratios and then mixed with dental glass powder and other essential components to separately fabricate three groups of bio-based RBS ISDSs featuring low diluent content and low double-bond density (groups 1–3; Supplementary Table S1).

#### 4.1.2 Preference of ISDS

Commercially available 3M ESPE Clinpro™ Sealant (Clinpro), which is the dental sealant prevalently used in clinical treatments and mainly comprises bis-GMA and TEGDMA, was chosen as the control material. Three important property indicators that are relevant to the sealing effect, namely, fluidity, double-bond conversion rate, and hydrophilicity of the dental sealants, were analyzed to determine the optimum group with the most favorable matrix/diluent ratio. Through the measurements of the rheological viscosity and contact angle test of the uncured sealant liquid on the bovine enamel surface, compared with Clinpro, the two ISDS groups demonstrated a comparable or even superior fluidity (Figure 2A) and a similar wetting ability on the enamel surface (Figure 2B), although the percentage of diluents (13%–27%) in the ISDS was significantly lower than that in Clinpro (more than 40%) because the viscosity of IBMEDS was notably lower than that of bis-GMA (Kim et al., 2006; Song et al., 2019). This must be considered because the exposure of complete RBS structures to a moist oral environment for an extended time after curing would gradually damage the structure because of the leaching of unreacted resin monomers and significantly decelerate the hydrolysis of the resin material, thus adversely affecting its longevity (Ferracane, 2006; Kerby et al., 2009). Accordingly, the double-bond conversion (or residue) rate and hydrophilicity of the sealant materials were examined. Based on the FT-IR results (Figure 2C) and the water contact angle on the surface of the resin specimen (Figure 2D), despite group 2 exhibiting a slightly higher viscosity (0.518 Pas) than Clinpro (0.475 Pas) at a shear rate of 1 s^-1^, neither the double-bond residue rate nor the water contact angle demonstrated a statistical difference (*p* > 0.05) compared with Clinpro, indicating that group 2 should have a comparable structural stability to that of Clinpro after photopolymerization, which can be attributed to the lower C=C double-bond density and the lesser amount of hydrophilic isosorbide cores in group 2 than in group 3 (Herrera-González et al., 2014; Berlanga et al., 2017; Fenouillot et al., 2010). Based on the comprehensive consideration of the fluid wettability of the uncured liquid sealant and the hydrolysis resistance of the resin structure, group 2, with an 85:15 (wt%) component ratio of IBMEDS to IBM, was finally selected as the optimal group among the three groups of ISDSs. Subsequently, for ISDS group 2, a series of comparative studies with Clinpro were conducted to comprehensively evaluate its clinical application potential.

### 4.2 Cytotoxicity evaluation of the fluoride-containing ISDS

The biosafety of dental sealants should be valued, particularly since the majority of the patients using them are children under 13 years old (World Health Organization, 2013; Manoj et al., 2018). In addition, RBSs must be equipped with a fluoride-release capacity to exert a local anti-caries effect (Prabhakar et al., 2012). Therefore, tetrabutylammonium tetrafluoroborate, which is an organic fluoride-containing compound, was selected and added to the ISDS group 2 in different proportions (Supplementary Table S2) to evaluate its cytotoxic effects. As shown in Figure 3G and Supplementary Table S2, the cell viabilities corresponding to the ISDSs with various fluoride reagent contents (all above 50%) were markedly higher than those of Clinpro (7.69%) (*p* < 0.0001); this result was also supported by the density of living cells under fluorescence microscopy (Figures 3A–F). This phenomenon can be because ISDS is a material specifically constructed from bio-based isosorbides (Gregorí Valdés et al., 2018; Kang et al., 2014). Because of its excellent biocompatibility, ISDS not only improved the controversial biocompatibility issues faced in traditional RBSs but also contributed to reducing or avoiding potential damage to oral or physical health after treatment. After weighing the balance between the anti-caries ability and cell toxicity of the material, the ISDS with a 4% content of the fluoride reagent was selected for further studies regarding the long-term stability, microleakage, and anti-caries capability of the material.

### 4.3 Long-term stability estimation of fluoride-containing ISDS

The long-term stability of pit and fissure sealants is a critical clinical indicator. Because of the polymerization shrinkage stress during the photo-curing of the RBS, the bonding ability of the resin material to the tooth interface is weakened, leading to marginal gaps or micro-cracking between them, which consequently causes shedding of the sealing material (Soares et al., 2017; Mehrabkhani et al., 2015). In addition, the damp oral environment poses a risk to RBS because its structural integrity is compromised by the hydrolysis and leaching of residual monomers from the material (Huang et al., 2014; Yuan et al., 2015). Therefore, four indicators, namely, polymerization volumetric shrinkage (PS), shear BS, WS, and SL of the 4% fluoride-containing ISDS were measured to comprehensively estimate its long-term stability.

First, linear shrinkage behaviors of Clinpro and fluoride-containing ISDSs were observed using linear variable differential transformers (LVDT). The linear PS rate (1.745%) of the fluoride-containing ISDS was significantly lower than that of Clinpro (2.640%) by approximately 34% (*p* < 0.0001), as shown in Figure 4A. Furthermore, the double-bond density of the ISDS group was calculated to be approximately 2.17 × 10^21^/g, whereas that of Clinpro was approximately 3.25 × 10^21^/g (Liu et al., 2013; He et al., 2019), indicating that compared to Clinpro, the reduction in curing shrinkage of this constructed fluoride-containing ISDS was nearly consistent with the decrease rate of the C=C density. Additionally, according to the results of the measured BS, the fluoride-containing ISDS (16.84 ± 2.22 MPa) formed a significantly stronger mechanical bond with the tooth enamel than Clinpro (13.10 ± 2.45 MPa) after curing (*p* < 0.01) (Figure 4A) because of the low PS of the ISDS group, which effectively reduced the weakening effect of the bond strength between the RBS material and enamel caused by the shrinkage stress during polymerization and ultimately presented a superior binding effect. Meanwhile, the fluoride-containing ISDS exhibited lower WS (*p* < 0.01) and SL (*p* < 0.0001) than that of Clinpro (Figure 4B), which may be because the isosorbide group, which had a larger volume than that of the triglycol group in TEGDMA and a weaker hydrophilicity than that of the hydroxyl group in bis-GMA, restricted the dissolution of unreacted monomers and hydrogen bonds between the materials and water (Ferracane, 2006; Kerby et al., 2009; Sideridou et al., 2003). The aforementioned results demonstrate apparent advantages of the fluoride-containing ISDS with respect to the following three aspects: low polymerization shrinkage, high shear bonding strength, and low WS and SL, indicating that its structure would remain more intact in a humid oral environment, thus ensuring the long-term stability of RBS after the sealing treatment. Microleakage tests will be performed to further evaluate the marginal adaptability of the ISDS tooth enamel.

### 4.4 Microleakage and enamel marginal adaptation studies of fluoride-containing ISDS

Microleakage is the main reason for the clinical failure of RBS sealing treatments (Mehrabkhani et al., 2015; Meller et al., 2015). Microleakage is also an important indicator for evaluating whether a good RBS–enamel surface bond is formed and predicting the clinical service life of RBSs. The results of this study indicated that there was no significant difference in the microleakage scores between the ISDS group and the Clinpro control group (*p* > 0.05) (Supplementary Table S3; Figure 5D), and no microleakage occurred in the vast majority of teeth individually sealed by the two groups (Figures 5A–C), indicating that both groups showed excellent marginal sealing effects on the tooth surface. However, based on the SEM images, under conditions of low acid etching, the constructed fluoride-containing ISDS bonded better with the tooth enamel and formed a more continuous interface than Clinpro (Figures 5E–G), and it could penetrate into the exposed enamel pores after strong acid etching to form long and deep resin tags, as shown in Figure 5J. Sparse resin tags were inserted into the demineralized enamel pores (Figure 5I), forming microgaps between the material layer and enamel, which were larger for the Clinpro group than for the ISDS group (*p* < 0.001) (Figure 5H). The fluoride-containing ISDS demonstrated a superior bonding effect to enamel than Clinpro, although there was no significant difference in the anti-microleakage properties displayed by these two materials. These results emphasized the advantages of the low C=C double-bond density and low PS of this ISDS and provided more intuitive evidence for its higher BS, which is supported by the findings of Petrovic et al. (2010) regarding the incremental placement technique for shrinkage stress reduction.

### 4.5 Preliminary evaluation of the caries-resistant performance of the fluoride-containing ISDS

**FIGURE 6:**
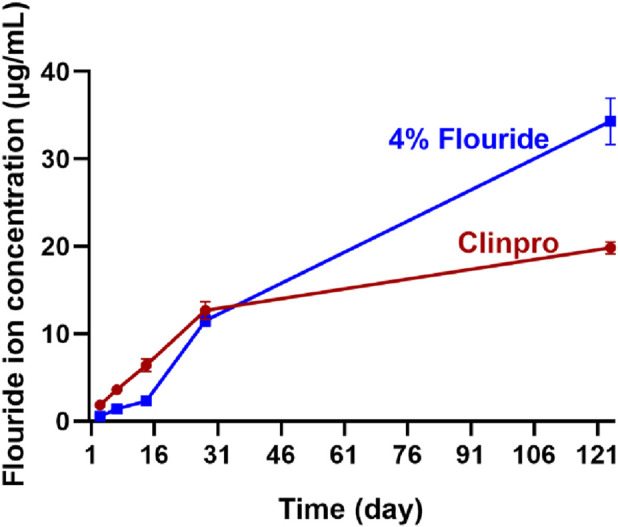
Concentration of the cumulative fluoride ion release of the 4% fluoride-containing ISDS and Clinpro.

Fluoride-containing RBSs interfere with caries by releasing fluoride ions to reduce enamel demineralization and enhance remineralization (Kaga et al., 2014; Thuy et al., 2008), whereas a ‘burst effect’ easily appears during the fluoride release of certain types of clinically used RBSs, which negatively impacts the short-term safety of periodontal cells, along with the long-term caries-resistance of RBSs (Attar and Turgut, 2003). As a result, assessing the anti-caries ability of fluoride-containing RBSs is critical for analyzing their long-term fluoride-release behavior. Studying the fluoride-release behavior of the 4% fluoride-containing ISDS for longer than 4 months demonstrated that our material did not present the ‘burst effect’ in the early stage (Figure 6), which may be because the selected tetrabutylammonium tetrafluoroboron fluorine-release reagent is an organic molecule with poor water solubility and because of the reversible hydrolysis of BF_4_^−^, leading to the slow formation and release of free fluoride ions (Archer et al., 2005). This phenomenon demonstrates the possibility that this newly constructed fluoride-containing ISDS has a long-term caries-resistant effect while maintaining good biocompatibility.

Meanwhile, the concentration of fluoride ions released by the ISDS group was one-third of that released by the Clinpro group, which was significantly lower than the latter during the first 14 days (*p* < 0.0001), which is possibly related to the water absorption properties of the RBS material (Fúcio et al., 2016). Compared with the fluoride-containing isosorbide-based ISDS, the hydrophilic hydroxyl groups of bis-GMA in the Clinpro group had stronger interactions with water, leading to the presence of more ample water molecules inside the Clinpro materials and a significantly stronger tendency to dissolve and promote the BF_4_^−^ hydrolysis of the tetrabutylammonium tetrafluoroboron fluorine-release reagent. After 28 days, the water absorption values of the resin specimens in the two RBS groups successively reached saturation and caused gradual and consistent hydrolysis of BF_4_^−^ hydrolysis. When the placement time of the two RBS specimen groups in water was extended to 4 months, the concentration of the released fluoride ions in the ISDS group was 1.7 times that of Clinpro, and the density of the cross-linking network of materials was believed to be the main factor affecting fluorine ion release at that time (Olmos-Olmos et al., 2021). Because of the lower number of C=C double bonds in the ISDS group, the cross-linking network that was formed had a lower density than that of Clinpro, and upon undergoing long-term water immersion, the pores between the polymer chains expanded further because of a certain extent of material swelling (Moszner et al., 2008b), thus facilitating the release of more fluoride ions. Compared with Clinpro, the fluorine ions released in a slow and uniform manner by the ISDS not only avoid the potential harm to oral cells that may be caused by the high concentration of fluoride ions in the short-term but also introduce the potential to demonstrate a better caries-resistant effect over a longer period after treatment.

## 5 Conclusion

In summary, regarding the drawbacks of the polymerization, volumetric shrinkage, and poor biocompatibility of RBS for clinical use, we improved the traditional bis-GMA/UDMA/TEGDMA structural system of RBS, selected bio-based isosorbide as the raw material, and designed and prepared the IBMEDS matrix and IBM diluent monomers, eventually constructing a novel fluoride-containing ISDS with all the bio-based components. This bio-based ISDS not only infiltrates and seals pits and fissures similarly to Clinpro for clinical utilization but also demonstrates the following characteristics and performance: (1) superior long-term stability, which was manifested by the lower photo-curing volumetric shrinkage, water sorption and solubility, and higher shear bond strength than Clinpro. (2) The cell viability was greater than 65%, indicating the excellent biocompatibility of this isosorbide-based RBS and meeting the “no cytotoxicity” requirements of the ISO standards. (3) A more favorable and effective marginal adaption, namely, more continuous bonding interfaces and narrow microgaps formed between the ISDS and tooth enamel after photopolymerization. (4) A significantly slower and more permanent release of fluoride ions showed that this ISDS demonstrated the potential effect of long-term caries-resistance. Based on these unique advantages, the constructed fluoride-containing ISDS is promising for avoiding the biological hazard of existing RBSs for children and adolescents, reducing the risk of secondary caries and microleakage arising from PS after sealing treatment, and improving the stability of fluoride release and the anti-caries effect, thus demonstrating application prospects and a clinical significance.

## Data Availability

The raw data supporting the conclusions of this article will be made available by the authors, without undue reservation.
