# Supplementary material for: Development and pre-clinical evaluation of an isosorbide-based pit and fissure sealant achieving low polymerization shrinkage and high biocompatibility
Source: Front Bioeng Biotechnol. 2026 Apr 1;14:1730749. doi: 10.3389/fbioe.2026.1730749 (PMC13079693; doi:10.3389/fbioe.2026.1730749)
Supplement: Supplementary file 1 [file Supplementaryfile1.docx]

Supporting Information

Research and pre-clinical evaluation of Isosorbide-based pit and fissure sealant provided low polymerization shrinkage and high biocompatibility

**
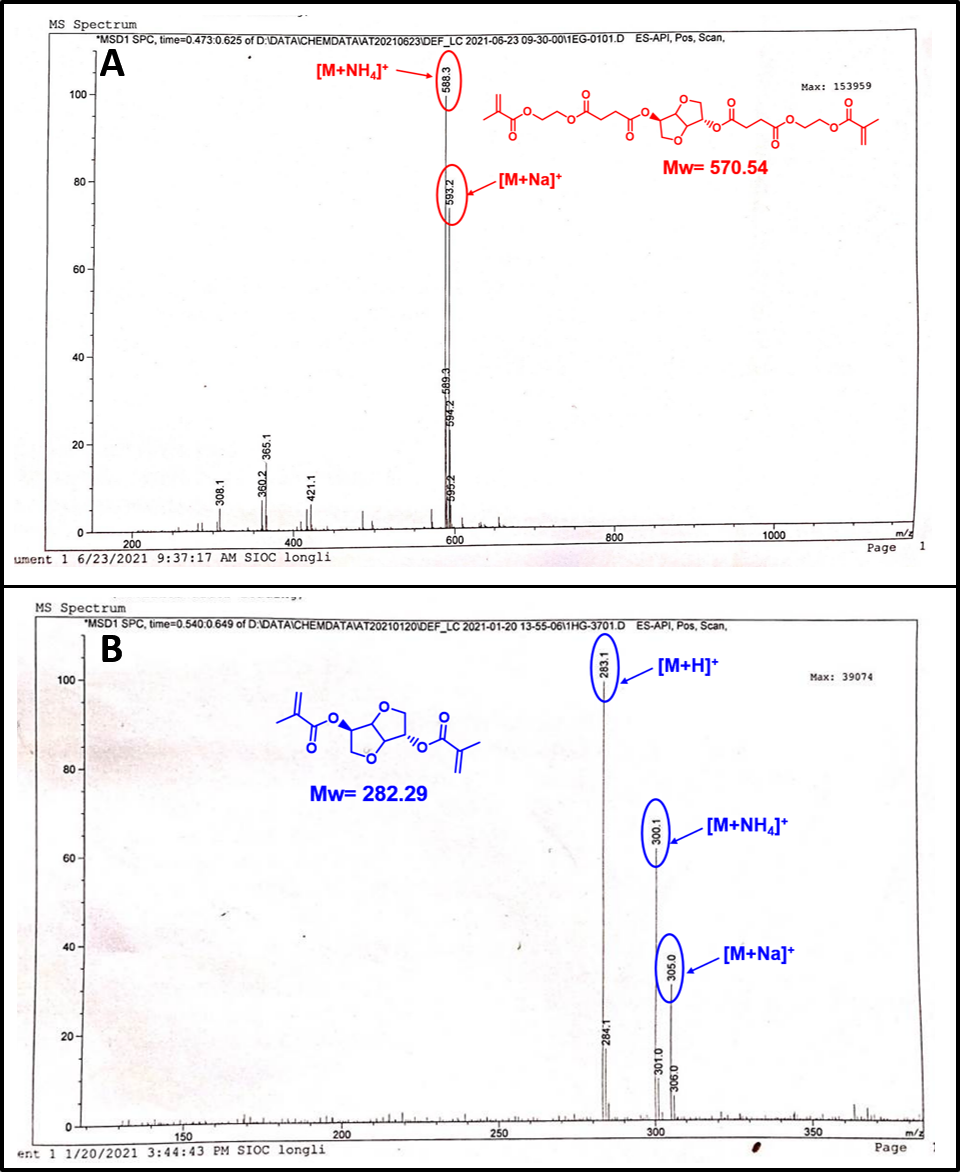
**

**Fig. S1** ESI-MS spectrum of (A) IBMEDS molecule and (B) IBM molecule.

**

**

**Fig. S2** FT-IR spectrum of silane-modification of SiO_2_.

**Table S1 Composition of experimental groups (wt%) for ISO sealant**

| **Group** | **IBMEDS** | | **IBM** | **glass powder** | **CQ** | **DMAEMA** | **Ratio of IBMEDS/IBM** |
| --- | --- | --- | --- | --- | --- | --- | --- |
| **Group1** | 89 | 0 | | 10 | 0.2 | 0.8 | 10/0 |
| **Group2** | 75.65 | 13.35 | | 10 | 0.2 | 0.8 | 8.5/1.5 |
| **Group3** | 62.3 | 26.7 | | 10 | 0.2 | 0.8 | 7/3 |

**Table S2** **L929 cell viability (%) of different tetrabutylammonium tetrafluoroborate content (wt%) contained ISO sealant**

| Group | 0 Fluoride | 2% Fluoride | 4% Fluoride | 6% Fluoride |
| --- | --- | --- | --- | --- |
| Fluoride reagent content (wt%) | 0 | 2 | 4 | 6 |
| L929 cell viability (%) | 84.9±2.63 | 79.9±3.83 | 67.1±3.21 | 53.7±1.59 |

**Table S3 Microleakage scores of Clinpro and** **fluoride contained ISO sealant**

| Group | Microleakage score（%） | | | |  |
| --- | --- | --- | --- | --- | --- |
|  | 0 | 1 | 2 | 3 | *p* value |
| Clinpro | 8 (80) | 2 (20) | 0 (0) | 0 (0) | 0.474 |
| 4% flouride | 10 (100) | 0 (0) | 0 (0) | 0 (0) |  |
